# Riociguat in patients with chronic thromboembolic pulmonary hypertension: results from an early access study

**DOI:** 10.1186/s12890-017-0563-7

**Published:** 2017-12-28

**Authors:** Vallerie V. McLaughlin, Pavel Jansa, Jens E. Nielsen-Kudsk, Michael Halank, Gérald Simonneau, Ekkehard Grünig, Silvia Ulrich, Stephan Rosenkranz, Miguel A. Gómez Sánchez, Tomás Pulido, Joanna Pepke-Zaba, Joan Albert Barberá, Marius M. Hoeper, Jean-Luc Vachiéry, Irene Lang, Francine Carvalho, Christian Meier, Katharina Mueller, Sylvia Nikkho, Andrea M. D’Armini

**Affiliations:** 1 University of Michigan Health System, 1011 Cornwell Pl, Ann Arbor, 48104 USA; 2 Cardiology and Angiology Department, General University Hospital, Prague, Czech Republic; 3 Department of Cardiological Medicine, Aarhus University, Aarhus, Denmark; 4 University Hospital Dresden, Dresden, Germany; 5 Hôpital Bicêtre, Université Paris-Sud, Laboratoire d’Excellence en Recherche sur le Médicament et Innovation Thérapeutique, and INSERM Unité 999, Paris, France; 6 Thoraxclinic, University Hospital Heidelberg, Heidelberg, Germany; 7 Clinic of Pulmonology, University Hospital Zurich, Zurich, Switzerland; 8 Department III of Internal Medicine, Cologne University Heart Center, Cologne, Germany; 9 Unidad de I. Cardiaca e Hipertensión Pulmonar, Hospital Universitario 12 de Octubre, Madrid, Spain; 10 Instituto Nacional de Cardiología, Mexico City, Mexico; 11 National Pulmonary Vascular Diseases Unit, Papworth Hospital, Cambridge, UK; 12 Department of Pulmonary Medicine, Hospital Clínic-IDIBAPS, University of Barcelona, and Biomedical Research Networking Center on Respiratory Diseases, Madrid, Spain; 13 Clinic for Respiratory Medicine, Hannover Medical School, Hannover, Germany; 14 Département de Cardiologie, Hôpital Erasme, Université Libre de Bruxelles, Brussels, Belgium; 15 Allgemeines Krankenhaus der Stadt Wien, Medizinische Universität Wien, Wien, Austria; 16 Global Development, Bayer SA, São Paulo, Brazil; 17 Global Medical Affairs, Bayer AG, Berlin, Germany; 18 Bayer AG, Wuppertal, Germany; 19 Global Clinical Development, Bayer AG, Berlin, Germany; 20 Division of Cardiothoracic Surgery, Foundation “IRCCS Policlinico San Matteo”, University of Pavia School of Medicine, Pavia, Italy

**Keywords:** Riociguat, Chronic thromboembolic pulmonary hypertension, Early access study

## Abstract

**Background:**

Following positive results from the Phase III CHEST-1 study in patients with inoperable or persistent/recurrent chronic thromboembolic pulmonary hypertension (CTEPH), the Phase IIIb CTEPH early access study (EAS) was designed to assess the safety and tolerability of riociguat in real-world clinical practice, as well as to provide patients with early access to riociguat before launch. Riociguat is approved for the treatment of inoperable and persistent/recurrent CTEPH.

**Methods:**

We performed an open-label, uncontrolled, single-arm, early access study in which 300 adult patients with inoperable or persistent/recurrent CTEPH received riociguat adjusted from 1 mg three times daily (tid) to a maximum of 2.5 mg tid. Patients switching from unsatisfactory prior pulmonary arterial hypertension (PAH)-targeted therapy (*n* = 84) underwent a washout period of at least 3 days before initiating riociguat. The primary aim was to assess the safety and tolerability of riociguat, with World Health Organization functional class and 6-min walking distance (6MWD) as exploratory efficacy endpoints.

**Results:**

In total, 262 patients (87%) completed study treatment and entered the safety follow-up (median treatment duration 47 weeks). Adverse events were reported in 273 patients (91%). The most frequently reported serious adverse events were syncope (6%), right ventricular failure (3%), and pneumonia (2%). There were five deaths, none of which was considered related to study medication. The safety and tolerability of riociguat was similar in patients switched from other PAH-targeted therapies and those who were treatment naïve. In patients with data available, mean ± standard deviation 6MWD had increased by 33 ± 42 m at Week 12 with no clinically relevant differences between the switched and treatment-naïve subgroups.

**Conclusions:**

Riociguat was well tolerated in patients with CTEPH who were treatment naïve, and in those who were switched from other PAH-targeted therapies. No new safety signals were observed.

**Trial registration:**

ClinicalTrials.org NCT01784562. Registered February 4, 2013.

**Electronic supplementary material:**

The online version of this article (10.1186/s12890-017-0563-7) contains supplementary material, which is available to authorized users.

## Background

Chronic thromboembolic pulmonary hypertension (CTEPH) is a form of pulmonary hypertension (PH) that results from obstruction of the pulmonary vasculature by residual organized thrombi. This leads to increased pulmonary vascular resistance (PVR), progressive PH, and ultimately death due to right ventricular failure [1–3]. Pulmonary endarterectomy (PEA), the gold-standard treatment for CTEPH, can potentially cure the condition [4, 5]. However, up to 40% of patients with CTEPH are considered technically inoperable, while up to 51% of patients develop persistent/recurrent PH after undergoing PEA [6–9].

Riociguat is a soluble guanylate cyclase (sGC) stimulator [10] that is approved for the treatment of inoperable and persistent/recurrent CTEPH. Riociguat has a dual mode of action, sensitizing sGC to endogenous nitric oxide (NO) by stabilizing NO–sGC binding, and directly stimulating sGC via a different binding site, independently of NO. This restores the NO–sGC–cyclic guanosine monophosphate (cGMP) pathway and increases generation of cGMP [10]. In the 16-week, randomized, double-blinded Phase III CHEST-1 study, riociguat was well tolerated and significantly improved a range of clinical endpoints in patients with inoperable and persistent/recurrent CTEPH, including 6-min walking distance (6MWD), PVR, *N-*terminal pro-hormone of brain natriuretic protein, and World Health Organization functional class (WHO FC) [11]. In an open-label extension, CHEST-2, improvements in 6MWD and WHO FC persisted at 2 years, with no new safety signals identified [12, 13].

The CTEPH early access study (EAS) was initiated to assess the safety and tolerability of riociguat using inclusion and exclusion criteria similar to those in CHEST-1, but adjusted to reflect more closely real-world clinical practice. The CTEPH EAS also provided early access to riociguat – after positive Phase III results and before final approval – for patients with inoperable CTEPH or persistent/recurrent PH after PEA who had an inadequate response to off-label treatments approved for pulmonary arterial hypertension (PAH), and who could not participate in another CTEPH trial.

## Methods

Eligible participants were 18–80 years old, with CTEPH that was deemed technically inoperable by an experienced surgeon/physician or persistent/recurrent PH after PEA, who were not satisfactorily treated and could not participate in another CTEPH trial. Patients were either treatment naïve or had previously received treatment with phosphodiesterase type 5 (PDE5) inhibitors, endothelin receptor antagonists (ERAs), or prostanoids. The study was carried out in accordance with Good Clinical Practice Guidelines and the Declaration of Helsinki. The study protocol was approved by the ethics committees of all participating centers and all patients gave their written informed consent.

This was an open-label, uncontrolled, single-arm, Phase IIIb long-term surveillance study (registered at ClinicalTrials.gov: identifier NCT01784562). The study consisted of three phases: an 8-week dose-adjustment phase; a maintenance phase that continued until riociguat was approved and commercially available in the patient’s respective country (except in the UK, where participation was limited to 18 months); and a safety follow-up phase, in which all patients who stopped study medication – including those who completed the study and transitioned to commercial riociguat – had a safety follow-up visit 30 days after discontinuation. During the dose-adjustment phase, riociguat dose was adjusted from a starting dose of 1 mg three times daily (tid) to a maximum of 2.5 mg tid according to systolic blood pressure and signs and symptoms of hypotension, as previously reported [11]. In cases of poor tolerability, a dose of 0.5 mg tid was permitted.

Patients not previously reaching their treatment goals (as judged by the investigator) with prior PAH-targeted therapies (PDE5 inhibitors, ERAs, or prostanoids) were switched to riociguat. All switched patients underwent a mandatory washout period (minimum 3 days) before initiating riociguat. Patients were permitted to initiate concomitant treatment with ERAs or prostanoids during the maintenance phase of the study (after the dose-adjustment phase) if the investigator considered it a medical requirement, but treatment with specific or non-specific PDE5 inhibitors, or NO donors, was not permitted.

The primary aim of the study was to assess the safety and tolerability of riociguat. Syncope was pre-defined as an adverse event (AE) of special interest in the study protocol. Events of syncope were reported as serious adverse events (SAEs) by the investigator and followed up with a questionnaire. In addition, clinical efficacy was assessed using WHO FC and optional assessment of 6MWD. Study visits were conducted every 2 weeks until Week 8 (dose-adjustment phase), then at Week 12 and at 12-week intervals thereafter (maintenance phase), with a safety follow-up visit 30 days after discontinuation for all patients who stopped study medication (safety follow-up phase).

All variables were analyzed descriptively in this open-label, non-comparative study. The statistical evaluation was performed using the SAS software package (release 9.2; SAS Institute Inc., Cary, NC, USA). The full analysis set included all patients who received at least one dose of study drug. Data were also analyzed in the subgroup of patients who switched from other PH medications to riociguat, defined as those who previously received an ERA, prostanoid, and/or PDE5 inhibitor. Baseline was defined as the last set of measurements taken before the first dose of riociguat.

## Results

**Fig. 1:**
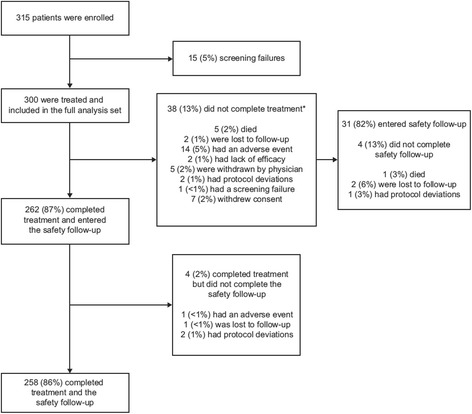
Patient disposition. *Patients who discontinued treatment prematurely were to enter the safety follow-up phase

In total, 300 patients, enrolled between March 2013 and December 2015, received riociguat treatment in the CTEPH EAS and were included in the full analysis set. Study treatment was completed by 262 patients (87%) (Fig. 1). Thirty-eight patients discontinued riociguat treatment during either the dose-adjustment or maintenance phase. The most frequent reason for discontinuation was an AE (*n* = 14). A further four patients discontinued during the safety follow-up phase, resulting in 258 patients (86%) completing the entire study. Baseline demographic and disease characteristics are shown in Table 1; most patients were female (62%), in WHO FC II/III (96%), and had inoperable CTEPH (72%). The median treatment duration was 47 weeks (range 0–121 weeks).

**Table 1:** Patient characteristics at baseline

Characteristic	Full analysis set (*n* = 300)	Switched patients (*n* = 84)^a^	Treatment-naïve patients (*n* = 216)
Sex, *n* (%)			
Female	185 (62)	55 (65)	130 (60)
Male	115 (38)	29 (35)	86 (40)
Age (mean ± SD), years	63.9 ± 12.5	65.5 ± 11.6	63.3 ± 12.7
Type of CTEPH, *n* (%)			
Inoperable	216 (72)	64 (76)	152 (70)
Persistent/recurrent	84 (28)	20 (24)	64 (30)
WHO FC, *n* (%)			
I	5 (2)	0	5 (2)
II	112 (37)	31 (37)	81 (38)
III	175 (58)	51 (61)	124 (57)
IV	8 (3)	2 (2)	6 (3)
6MWD (mean ± SD), m	374 ± 117^b^	389 ± 87^c^	369 ± 125^d^

*6MWD* 6-min walking distance, *CTEPH* chronic thromboembolic pulmonary hypertension, *ERA* endothelin receptor antagonist, *PDE5* phosphodiesterase type 5, *SD* standard deviation, *WHO FC* World Health Organization functional class

^a^Patients who previously received an ERA, prostacyclin, and/or PDE5 inhibitor, and who stopped this treatment before starting riociguat

^b^ *n* = 213; last observed value prior to start of study treatment

^c^ *n* = 52

^d^ *n* = 161

Of the 300 patients, 84 (28%) had switched to riociguat from prior PAH-targeted therapies at the discretion of the treating physician, with a median washout duration of 4 days (range 3–74 days). One patient had stopped PAH-targeted medication 74 days prior to starting treatment with riociguat, and was not taking PAH medication at screening, so can be considered as an outlier. In total, 58 patients (19%) switched from PDE5 inhibitors (most frequently sildenafil [14%]) and 44 patients (15%) switched from ERAs (most frequently bosentan [12%]; Table 2). In total, 24 patients (8%) were switched from combination therapy to riociguat; the most common combination therapy prior to switching was a PDE5 inhibitor plus an ERA.

**Table 2:** PAH-targeted therapy received prior to switching to riociguat

Therapy, *n* (%)	Full analysis set (*n* = 300)
Any prior therapy	84 (28)
Endothelin receptor antagonists	44 (15)
Ambrisentan	8 (3)
Bosentan	36 (12)
PDE5 inhibitors	58 (19)
Sildenafil	42 (14)
Tadalafil	16 (5)
Prostacyclins and prostacyclin analogues	7 (2)
Beraprost	1 (<1)
Iloprost	6 (2)
Combination therapy	24 (8)
Double therapy	23 (8)
Triple therapy	1 (<1)

*PAH* pulmonary arterial hypertension, *PDE5* phosphodiesterase type 5

At Week 12 (the first visit after the dose-adjustment phase), 237 of 263 available patients (90%) were receiving riociguat 2.5 mg tid, and no patients were receiving riociguat 0.5 mg tid. During the study, 286 patients (95%) started additional medication, most commonly cardiac therapy (65%) and drugs for gastrointestinal acid-related disorders (47%). PAH-targeted therapies were newly started or restarted by 42 patients (14%), including 18 patients (21%) from the switched subgroup (*n* = 84) and 24 patients (11%) from the treatment-naïve subgroup (*n* = 216). The majority of patients started/restarted PAH-targeted therapies due to worsening CTEPH. Thirty-six patients (12%) started ERAs during the study, and six (2%) started prostacyclins. Four patients (1%) started PDE5 inhibitor therapy during the study, of whom three discontinued riociguat on the same day, and one patient received PDE5 inhibitors for 1 day concomitantly with riociguat due to investigator error. In the switched subgroup 17 patients (20%) started ERAs, and one (1%) started prostacyclins during the maintenance phase. Two patients (2%) restarted PDE5 inhibitors on the same day as discontinuing riociguat. The most frequent reason for starting a new PAH-targeted medication was worsening PH, as determined by the investigator.

AEs were reported in 273 patients (91%) treated with riociguat (Table 3). The maximum severity of AEs experienced by individual patients was mild for 90 patients (30%), moderate for 109 patients (36%), and severe for 74 patients (25%). The most frequently reported AEs were dyspepsia (20%), dizziness (19%), headache (18%), and peripheral edema (18%) (Table 4). The most frequently reported SAEs were syncope (*n* = 17; 6%), right ventricular failure (*n* = 8; 3%), and pneumonia (*n* = 7; 2%).

**Table 3:** Summary of AEs during treatment with riociguat

AE, *n* (%)	Full analysis set (*n* = 300)	Switched patients (*n* = 84)^a^	Treatment-naïve patients (*n* = 216)
Any AE	273 (91)	76 (90)	197 (91)
Drug-related AEs	178 (59)	53 (63)	125 (58)
Serious AEs	89 (30)	22 (26)	67 (31)
Drug-related serious AEs	19 (6)	4 (5)	15 (7)
AEs leading to discontinuation of study medication	14 (5)	5 (6)	9 (4)
Deaths	5 (2)	0	5 (2)

*AE* adverse event, *PDE5* phosphodiesterase type 5, *ERA* endothelin receptor antagonist

^a^Patients who previously received an ERA, prostacyclin, and/or PDE5 inhibitor, and who stopped this treatment before starting riociguat

**Table 4:** AEs occurring in ≥10% of patients and AEs of special interest occurring during treatment with riociguat

	Full analysis set (*n* = 300)	Incidence per 100 patient-years
AE, *n* (%)		
Dyspepsia	60 (20)	27.5
Dizziness	56 (19)	26.0
Headache	54 (18)	29.4
Peripheral edema	54 (18)	23.0
Diarrhea	45 (15)	20.0
Nausea	43 (14)	18.8
Cough	38 (13)	16.6
Vomiting	34 (11)	16.6
Hypotension	29 (10)	12.4
Constipation	31 (10)	13.6
Gastroesophageal reflux disease	31 (10)	12.8
Nasopharyngitis	31 (10)	14.7
AE of special interest, *n* (%)		
Pre-syncope	10 (3)	4.1
Syncope	17 (6)	9.8

*AE* adverse event

During the washout phase between stopping prior non-satisfactory PAH-targeted therapy and initiation of riociguat (median duration 4 days, range 3–74 days), 11 of 84 patients (13%) in the switched subgroup experienced AEs. Eight of these AEs were mild in severity, and none was severe (Table 5). There were two SAEs during the washout phase: one event of possible syncope which started 3 days after discontinuing PDE5 inhibitor treatment (sildenafil) and resolved the same day; and one hospitalization resulting from septicemia which started 3 days after discontinuing ERA treatment (bosentan) and resolved 6 days later.

**Table 5:** Summary of AEs in switched patients during the washout phase of the study

AE, *n* (%)	Switched patients (*n* = 84)^a^
Any AE	11 (13)
Maximum intensity of any AE	
Mild	8 (10)
Moderate	3 (4)
Any serious AE	2 (2)
Deaths	0 (0)

*AE* adverse event, *ERA* endothelin receptor antagonist, *PDE5* phosphodiesterase type 5

^a^Patients who previously received an ERA, prostacyclin, and/or PDE5 inhibitor, and who stopped this treatment before starting riociguat

Five deaths (2%) were reported during the study (one case each of pleomorphic malignant fibrous histiocytoma, pneumonia, head injury, cardiac failure, and pulmonary embolism) and one additional patient died during the safety follow-up phase (due to cardiogenic shock as a result of pneumonia and worsening chronic heart failure). None of the deaths was considered by the investigator to be related to study medication.

All events of syncope (*n* = 17, 6%; Table 4) were reported as SAEs per definition; most were assessed as mild or moderate in intensity, and none led to permanent discontinuation of riociguat. Events of syncope were considered drug related in four patients; in two cases, the riociguat dose was unchanged, in one case the dose was reduced, and in one case riociguat was interrupted and later restarted. There was no association between dose adjustment of riociguat and events of syncope. Indeed, many events were associated with physical activity or were orthostatic in nature. In the majority of the cases there were no further episodes of syncope, or the events resolved after treatment of a concurrent illness or adjustment of concomitant medications.

All patients who experienced events of syncope or pre-syncope had concomitant diseases, and were receiving concomitant medications during the study, which may have increased the risk of an event. Furthermore, seven patients (26%) had previous episodes of syncope and three (11%) had previous episodes of dizziness. In terms of concomitant diseases, 23 patients (85%) had respiratory disorders, 16 (59%) had vascular disorders, and 14 (52%) had cardiac disorders. The most common concomitant medications were anticoagulants (*n* = 27, 100%), gastrointestinal protective drugs for acid-related disorders (*n* = 20, 74%), and diuretics (*n* = 18, 67%). Four patients (15%) received concomitant antihypertensive medications.

Dizziness was reported in 56 (19%) patients, eight (3%) patients experienced falls, and one patient (<1%) experienced orthostatic collapse. One patient had a fatal head trauma following an accidental fall; the patient had four previous episodes of falls, none of which was considered to be related to syncope or pre-syncope by the investigator, and no hypotension was reported in this patient.

Thirty-six hypotension-related events were reported in 32 patients (11%; a rate of 12.4 events per 100 patient-years), including 19 mild, 12 moderate, and five severe events. Nineteen events of hypotension occurred during the dose-adjustment phase, which led to dose reduction in four patients and drug withdrawal in one patient. Hypotension was classed as an SAE in four patients (1%), as the events required or prolonged hospitalization; riociguat treatment was interrupted in one patient and remained unchanged in the other three patients. All SAEs of hypotension were considered severe and had resolved by the end of the study.

Hemoptysis was reported in 11 patients (4%), of whom four (1%) were classified as having serious hemoptysis (moderate, *n* = 3; severe, *n* = 1). Two SAEs of hemoptysis were considered study drug related; in one case, no changes were made to the dose of riociguat and in the other case riociguat was withdrawn. All SAEs of hemoptysis had resolved by the end of the study.

Overall, the safety of riociguat was similar in patients who were switched from other PAH-targeted therapies and those who were treatment naïve (Table 3).

Four patients (1%) underwent balloon pulmonary angioplasty during the study, including one pre-planned procedure. Three of the procedures, consisting of between two and four interventions, were considered successful by the investigators, whereas in one patient stress cardiomyopathy was observed, which had not resolved by the end of the study. The patient who experienced stress cardiomyopathy was in WHO FC II at both baseline and Week 12, and had a consistent 6MWD >500 m, indicating no serious deterioration.

Assessment of 6MWD was optional during the CTEPH EAS, and therefore data were not available for all patients. The available data are summarized in Table 6. At baseline, mean ± SD 6MWD was 374 ± 117 (*n* = 213), and switched patients had numerically higher 6MWD compared with treatment-naïve patients (389 ± 87 m versus 369 ± 125 m). The percentage of patients in WHO FC I/II/III/IV at baseline was 2%/37%/58%/3% (*n* = 300) (Table 7). In patients who had a 6MWD measurement at Week 12, mean ± SD change from baseline was +33 ± 42 m (*n* = 130; absolute value at Week 12 was 416 ± 111 m, *n* = 153) (Table 6). After 12 weeks of treatment (*n* = 264), WHO FC had improved in 58 patients (22%), remained stable in 193 (73%), and worsened in 13 (5%) (Table 7). Improvements in 6MWD and WHO FC were seen in both treatment-naïve and switched patients (Tables 6 and 7).

**Table 6:** Change from baseline in 6MWD (optional assessment)

Timepoint	Full analysis set		Switched patients^a^		Treatment-naïve patients	
	*n*	Change from baseline (mean ± SD), m	*n*	Change from baseline (mean ± SD), m	*n*	Change from baseline (mean ± SD), m
Dose-adjustment phase						
Week 2	75	+20 ± 42	22	+8 ± 48	53	+25 ± 38
Week 4	77	+34 ± 39	19	+36 ± 31	58	+34 ± 42
Week 6	72	+41 ± 49	20	+30 ± 39	52	+45 ± 53
Week 8	93	+30 ± 70	21	+26 ± 47	72	+31 ± 76
Maintenance phase						
Week 12	130	+33 ± 42	32	+28 ± 39	98	+34 ± 43
Week 24	105	+30 ± 63	20	+32 ± 45	85	+29 ± 67
Week 36	93	+32 ± 59	24	+37 ± 44	69	+31 ± 64
Week 48	62	+42 ± 60	13	+36 ± 68	49	+43 ± 59

*6MWD* 6-min walking distance, *ERA* endothelin receptor antagonist, *PDE5* phosphodiesterase type 5, *SD* standard deviation

^a^Patients who previously received an ERA, prostacyclin, and/or PDE5 inhibitor, and who stopped this treatment before starting riociguat

**Table 7:** Change from baseline in WHO FC

Timepoint	Full analysis set		Switched patients^a^		Treatment-naïve patients	
	*n*	Improved/stabilized/ worsened (%)	*n*	Improved/stabilized/ worsened (%)	*n*	Improved/stabilized/ worsened (%)
Dose-adjustment phase						
Week 2	293	8/90/2	82	5/94/1	211	9/89/3
Week 4	292	13/84/2	81	12/86/1	211	14/83/3
Week 6	289	15/83/2	79	11/87/1	210	16/82/2
Week 8	284	19/79/2	78	17/82/1	206	20/78/2
Maintenance phase						
Week 12	264	22/73/5	70	21/76/3	194	22/72/6
Week 24	208	25/70/5	52	17/79/4	156	28/67/5
Week 36	162	30/69/1	43	23/77/0	119	32/66/2
Week 48	114	29/69/2	28	21/79/0	86	31/66/2

*ERA* endothelin receptor antagonist, *PDE5* phosphodiesterase type 5, *WHO FC* World Health Organization functional class

^a^Patients who previously received an ERA, prostacyclin, and/or PDE5 inhibitor, and who stopped this treatment before starting riociguat

## Discussion

The open-label, uncontrolled CTEPH EAS provided further information on the safety and clinical efficacy of riociguat in patients with CTEPH, and gave access to riociguat for patients who could not participate in another clinical trial. The results of this study were in agreement with the results of the Phase III CHEST-1 study and the CHEST-2 long-term extension [11–13], and showed that riociguat is well tolerated in patients with CTEPH.

As riociguat is a vasodilator, potential side effects include hypotension and hypotension-related disorders. The rate of hypotension in patients with CTEPH has previously been shown to decrease with increasing riociguat treatment duration. At the end of the 16-week CHEST-1 trial, the rate of hypotension was 31.2 events per 100 patient-years, while after 2 years of CHEST-2 (median treatment duration 116 weeks) the rate of hypotension had fallen to 4.0 events per 100 patient-years [13]. As the median treatment duration in the CTEPH EAS (47 weeks) lies between the durations in CHEST-1 and CHEST-2, the recorded rate of hypotension of 12.4 events per 100 patient-years appears to be in the expected range.

Syncope is a known symptom of PH, associated with reduced central perfusion. Events of syncope, as an outcome-related symptom of interest to treating physicians, were recorded as AEs of special interest in the CTEPH EAS, and were assessed using a targeted questionnaire. In the previous controlled study, CHEST-1, syncope was not associated with riociguat treatment. In agreement with this, the questionnaire and available information in the CTEPH EAS showed no direct association between the administration of riociguat and occurrence of syncope. Although the rate of syncope in the CTEPH EAS (9.8 events per 100 patient-years) was higher than the rates of syncope observed in the riociguat arm of CHEST-1 and the CHEST-2 long-term extension (7.8 events per 100 patient-years and 5.2 events per 100 patient-years, respectively), it was lower than the rate observed in the placebo arm of the CHEST-1 study (15.1 events per 100 patient-years) [13]. Moreover, the proportions of patients experiencing syncope with riociguat in CHEST-1 and -2 and the CTEPH EAS (2%, 10% and 6%, respectively) were lower than in the international CTEPH registry, in which 13.7% of patients experienced syncope [9]. Data on the rate of syncope in CTEPH patients are lacking, and syncope has not been investigated as an event of special interest in other trials such as the BENEFiT study [14]. The results of the CTEPH EAS suggest that syncope and pre-syncope may occur in patients with CTEPH with many of the reported cases associated with physical exertion or of orthostatic nature, or in context with underlying conditions or concomitant medications. However, it should also be noted that there are many potential causes for syncope in the relatively elderly patient population enrolled in this study (mean age 64 years versus 59 years in the CHEST-1 study [11]).

Overall, we found that the safety profile of riociguat in this study was consistent with that observed in CHEST-1 and CHEST-2 [11–13], with the usual vasodilatory effects, and no new safety signals were reported. The safety profile was also consistent with that seen in patients with PAH in the Phase III PATENT study in patients with PAH [15].

As the main aim of the CTEPH EAS was to assess the safety and tolerability of riociguat, and 6MWD assessments were therefore optional, only half of the patients recorded 6MWD at Week 12. An improvement in 6MWD of +33 m was observed at Week 12, while the improvement after 1 year was +37 ± 72 m (*n* = 43). However, these results need to be interpreted with caution because of the exploratory nature of the efficacy assessments in the CTEPH EAS.

Although riociguat is the only approved therapy for inoperable and persistent/recurrent CTEPH, off-label treatment with drugs approved for PAH, including PDE5 inhibitors, ERAs, and prostanoids, is common [9, 16–18]. In this study, 84 patients (28%) switched to riociguat monotherapy from previous treatment with PAH-approved therapies on which they had shown an insufficient clinical response. Of these patients, 24 (8%) were previously receiving combination therapy, including one patient on triple therapy. While it should be noted that in order to minimize risks to the patient this would not be the usual approach to changing treatment regimens in clinical practice, the safety and tolerability of riociguat in patients who switched was similar to that in patients who were treatment naïve, regardless of their previous PAH-targeted treatment regimen. Furthermore, there were no apparent safety issues associated with the treatment-free washout period (median duration 4 days, range 3–74 days). Although 11 patients (13%) experienced AEs during this phase, the majority of the cases were mild (10%). In addition, there were no relevant differences in change from baseline in 6MWD and WHO FC between patients in the switched and treatment-naïve subgroups.

Baseline real-world data have been published from national and international registries of patients with CTEPH [7–9, 16–19], showing similar demographic characteristics to those of patients in the CTEPH EAS (mean age, 57–61 versus 64 years, respectively) [7, 8, 18, 19] and including 46–60% versus 62% female patients, respectively [7–9, 16–19]. There were, however, differences in baseline exercise and functional capacity between patients in the CTEPH EAS compared with registries. For example, 39% of patients in the CTEPH EAS were in WHO FC I or II at baseline compared with 9–23% of those in the registries [9, 16, 18]. Similarly, mean 6MWD at baseline was higher for patients in the CTEPH EAS compared with patients in the registries (374 m versus 239–341 m) [7–9, 16–19]. In addition, fewer patients in the CTEPH EAS were previously receiving PAH-targeted therapies compared with those enrolled in the registries (28% versus 29–90%). Baseline demographic characteristics in the CTEPH EAS were also similar to those in the CHEST-1 study (female patients 62% versus 66%, respectively), although disease severity in terms of WHO FC I/II (39% versus 32%) and 6WMD (374 m versus 347 m) was slightly worse in the CHEST-1 study [11]. Unlike patients in the CTEPH EAS, however, patients in CHEST-1 were excluded if they had received prior PH therapy within 3 months before study entry.

The limitations of the CTEPH EAS study, including the open-label, non-comparative design, are common to all long-term safety studies. In addition, the use of concomitant therapy in the study means that the safety and efficacy findings cannot unequivocally be attributed to riociguat. However, there was a relatively low rate of new PAH-targeted concomitant therapies throughout the study (14%). It should also be noted that assessment of 6MWD in the CTEPH EAS was optional, leading to a potential negative bias and relatively low patient numbers. Nevertheless, open-label, non-comparative studies such as the CTEPH EAS are important to bridge the gap between Phase III studies and real-world data from registries.

## Conclusions

In conclusion, riociguat was well tolerated in patients with CTEPH, with no new safety signals observed compared with other riociguat trials. Furthermore, no relevant differences in the safety profile were detected in treatment-naïve patients and those switched from other PAH-targeted therapies. Improvements in 6MWD and WHO FC were also observed. The data in the CTEPH EAS support the previous evidence for riociguat as a long-term treatment option for patients with CTEPH.

## Additional file

Additional file 1: Institutional Ethics Committees. (DOCX 18 kb)

## Abbreviations

6MWD: 6-min walking distance
AE: Adverse event
cGMP: Cyclic guanosine monophosphate
CTEPH: Chronic thromboembolic pulmonary hypertension
EAS: Early access study
ERA: Endothelin receptor antagonist
NO: Nitric oxide
PAH: Pulmonary arterial hypertension
PDE5: Phosphodiesterase type 5
PEA: Pulmonary endarterectomy
PH: Pulmonary hypertension
PVR: Pulmonary vascular resistance
SAE: Serious adverse event
SGC: Soluble guanylate cyclase
tid: Three times daily
WHO FC: World Health Organization functional class
