# Supplementary material for: Riociguat in patients with chronic thromboembolic pulmonary hypertension: results from an early access study
Source: BMC Pulm Med. 2017 Dec 28;17:216. doi: 10.1186/s12890-017-0563-7 (PMC5745920; doi:10.1186/s12890-017-0563-7)
Supplement: Additional file 1: — Institutional Ethics Committees. (DOCX 18 kb) [file 12890_2017_563_MOESM1_ESM.docx]

**Additional file 1**

**Institutional Ethics Committees**

| **Trial unit** | **Trial unit reference** | **Country** | **Institutional Ethics Committee location** |
| --- | --- | --- | --- |
| 44001 | Not Available | Austria | Allgemeines Krankenhaus der Stadt Wien Universitätskliniken  Ethikkommission der Medizinischen Universität Wien und des AKH  Borschkegasse 8b/E 06  1090 Wien |
| 44003 | Not Available | Austria | Medizinische Universität Graz  Ethikkommission der Medizinischen Universität Graz Auenbruggerplatz 2  8036 Graz |
| 44002 | Not Available | Austria | Landeskrankenhaus - Universitätskliniken Innsbruck Ethikkommission der Medizinischen Universität Innsbruck  Innrain 43  6020 Innsbruck |
| 28001 | 28001 | Belgium | Hôpital Erasme/Erasmus Ziekenhuis  Comité Ethique/Ethisch Comité  Route de Lennik 808 Lenniksebaan  1070 BRUXELLES – BRUSSEL |
| 28002 | 28002 | Belgium | UZ Leuven Gasthuisberg  Commissie voor Medische Ethiek/klinisch Onderzoek E330  Herestraat 49  3000 LEUVEN |
| 26001 | Not Available | Canada | Sir Mortimer B. Davis Jewish General Hospital  Sir Mortimer B. Davis Research Ethics Offfice  Suite A-925  3755 Ch. Cote Ste-Catherine  H3T 1E2 Montreal |
| 26004 | Not Available | Canada | University of Ottawa Heart Institute  Ottawa Health Science Network-Research Ethics Board 725 Parkdale Avenue  Civic Box 411  K1Y 4E9 Ottawa |
| 58004 | Not Available | Switzerland | Kantonale Ethikkommission Bern  Postfach 56  3010 Bern |
| 58002 | Not Available | Switzerland | Kantonale Ethikkommission Zürich  Stampfenbachstrasse 121  8090 Zürich |
| 38001 | 38001 | Czech Republic | Eticka komise  Vseobecne fakultni nemocnice v Praze  Na Bojisti 1  128 08 Praha 2 |
| 10001 | Not Available | Germany | Kliniken der Medizinischen Hochschule Hannover  Ethik-Kommission  Carl-Neuberg-Str. 1  30625 Hannover |
| 10002 | Not Available | Germany | Universitätsklinikum Heidelberg  Ethikkommission der Med. Fakultät Heidelberg  Alte Glockengießerei 11/1  69115 Heidelberg |
| 10011 | Not Available | Germany | Universitätsklinikum Regensburg  Ethikkommission an der Universität Regensburg Dienstgebäude "Altes Finanzamt"  Raum 134, 1. OG.  Landshuter Straße 4  93047 Regensburg |
| 10005 | Not Available | Germany | Universitätsklinikum Köln  Ethikkommission der Medizinischen Fakultät  Gebäude 55  Kerpener Str. 62  50937 Köln |
| 10006 | Not Available | Germany | Medizinische Fakultät Carl Gustav Carus  Ethik-Kommission der Technischen Universität Dresden  Fetscherstraße 74  01307 Dresden |
| 10009 | Not Available | Germany | Ethikkommission der Bayerischen Landesärztekammer Mühlbaurstr. 16  81677 München |
| 10007 | Not Available | Germany | Ethik-Kommission der Ärztekammer Hamburg Weidestraße 122 b  22083 Hamburg |
| 10012 | Not Available | Germany | Ethik-Kommission bei der Ärztekammer des Saarlandes Faktoreistr. 4  66111 Saarbrücken |
| 10008 | Not Available | Germany | Universitätsklinikum Leipzig AöR  Ethik-Kommission an der Medizinischen Fakultät der Universität Leipzig Haus: Karl-Sudhoff-Institut  Käthe-Kollwitz-Straße 82  04109 Leipzig |
| 10003 | Not Available | Germany | Universitätsklinikum Giessen und Marburg  Ethik-Kommission des FB Medizin  Alte Frauenklinik  Klinikstr. 32  35392 Gießen |
| 35001 | Not Available | Denmark | Den Videnskabsetiske Komité for Region Midtjylland  Sundhedssekretariatet  Skottenborg 26  Postboks 21  DK-8800 Viborg |
| 24004 | 24004 | Spain | Ciutat Sanitària i Universitaria de la Vall d'Hebron  Comité Étic d'Investigació Clínica  Planta 13-Hospital Maternoinfantil  Passeig de la Vall d'Hebron, 119-129  08035 Barcelona |
| 24001 | 24001 | Spain | Hospital Universitario 12 de Octubre  Comité Ético de Investigación Clínica.  Inst. de Investigación Hospital 12 de Octubre-Área de Gestión de Proyectos-Unidad Admin. CEIC  Centro de Actividades ambulatorias. Bloque D, Pl. 6  Av. de Córdoba, s/n  28041 Madrid |
| 24002 | 24002 | Spain | Hospital Clínic i Provincial de Barcelona  Comité Ético de Investigación Clínica  Agencia de Ensayos Clínicos. Servicio de Farmacia Planta 0, Escalera 6B  C/ Villarroel, 170  08036 Barcelona |
| 24003 | 24003 | Spain | Hospital Universitario Virgen del Rocío  Unidad de Gestión de Ensayos Clínicos. Area de investigación  Edificio Laboratorios 6ª planta  Avda. Manuel Siurot, s/n  41013 Sevilla |
| 16001 16003 16004 16006 16007 16009 16010 16011 | 16001  16003  16004  16006  16007  16009  16010  16011 | France | Comité de Protection des Personnes CPP Ile de France VIII  Hôpital Ambroise Paré  9 Avenue Charles de Gaulle  92100 BOULOGNE BILLANCOURT |
| 12001 12002 12003 12005 | 12001 12002 12003 12005 | United Kingdom | North West - Liverpool East  North West Centre of Research Ethics Committees  3rd Floor, Barlow House  4 Minshull Street  M1 3DZ Manchester |
| 22003 | Not Available | Italy | A.O.U. Ospedali Riuniti Trieste  CERU: COMITATO ETICO REGIONALE UNICO (since 01Oct2013)  33100 Udine |
| 22002 | Not Available | Italy | Azienda Policlinico Umberto I  COMITATO ETICO UNIVERSITA' LA SAPIENZA (since 01Oct2013)  c/o Policlinico Umberto I  Viale del Policlinico, 155  00161 Roma |
| 22001 | Not Available | Italy | Fondazione IRCCS Policlinico San Matteo  COMITATO ETICO AREA DI PAVIA (since 01Oct2013)  c/o IRCCS Policlinico San Matteo  Viale C. Golgi, 19  27100 Pavia |
| 20003 | Not Available | Japan | The University of Tokyo Hospital  Institutional Review Board  7-3-1, Hongo  Bunkyo-ku Tokyo 113-8655 |
| 32003 | 32003 | Mexico | Instituto Nacional de Ciencias Médicas y Nutricion  Comité de ética en investigación  del Instituto Nacional de Ciencias Medicas y Nutrición Salvador Zubirán Vasco de Quiroga No 15  Col. Sección XVI  14080 México, D.F. |
| 30001  30002  30003 | Not Available  Not Available  Not Available | Netherlands | Vrije Universiteit Medisch Centrum  METC VUmc  BS7, kamer H-565  Van der Boechorststraat 7  Postbus 7057  1007 MB AMSTERDAM |
| 42001  42003 | 42001  42003 | Portugal | Comissão de Ética para a Investigação Clínica  Parque da Saúde de Lisboa  Av. do Brasil, no. 53-Pav. 17-A  1749-004 Lisboa |
| 51001 | Not Available | Russian Federation | North-West Federal Medical Research Center  2, Akkuratova st.  197341 St. Petersburg |
| 34002 | Not Available | Sweden | Regionala Etikprövningsnämnden i Umea  Samverkanshuset  Universitetsområdet  90137 Umeå |
| 47001 47002 47003 47004 47006 | 47001  47002  47003  47004  47006 | Turkey | Ege Univer.Medical Faculty Clinical Trials Ethical Committee  Ege Universitesi Tip Fakultesi Dekanlik Binas 2. kat Bornova  35100 Izmir |
| 14010 | Not Available | United States | Columbia University Medical Center  Institutional Review Board  722 West 168th Street  4th Floor - Room 426  New York  New York 10032 |
| 14003 | Not Available | United States | Chesapeake IRB  7063 Columbia Gateway Drive  Suite 110  Columbia  Maryland 21046-3403 |
| 14020 | Not Available | United States | University of Texas Southwestern Medical Center Institutional Review Board  5323 Harry Hines Boulevard  Dallas  Texas 75390 |
| 14006  14009 | Not Available  Not Available | United States | Cleveland Clinic  Cleveland Clinic Foundation IRB  9500 Euclid Avenue  Cleveland  Ohio 44195 |
| 14004 | Not Available | United States | Boston University School of Medicine  BUMC Institutional Review Board  500 Harrison Avenue  3rd Floor, Suite 300  Boston  Massachusetts 02118 |
| 14008 | Not Available | United States | University of Michigan Health System  IRBMED  2800 Plymouth Road  Building 520, Room 3214  Ann Arbor  Michigan 48109 |
| 14001 | Not Available | United States | University of California Davis Medical Center  UC Davis IRB Administration  CTSC Building  2921 Stockton Boulevard  Suite 1400/Room 1429  Sacramento  California 95817 |
| 14021 | Not Available | United States | University of Maryland Hospital System  University of Maryland, Professional Schools Institutional Review Board  800 Baltimore Street  Suite 100  Baltimore  Maryland 21201 |
| 14018 | Not Available | United States | Rhode Island Hospital  Committee for Protection of Human Subjects  Research Administration  593 Eddy Street  Aldrich Bldg. - 5th Floor  Providence  Rhode Island 02903 |
| 14002 | Not Available | United States | Colorado Multiple Institutional Review Board  Mail Stop F-490  PO Box 6508  Aurora  Colorado 80045 |
| 14005 14007 14011 14015 | Not Available  Not Available  Not Available  Not Available | United States | Western Institutional Review Board  1019 39th Avenue SE  Suite 120  Puyallup  Washington 98374-2115 |
